# Interactive Effects of Maternal Vitamin D Status and Socio-Economic Status on the Risk of Spontaneous Abortion: Evidence from Henan Province, China

**DOI:** 10.3390/nu14020291

**Published:** 2022-01-11

**Authors:** Shiqi Lin, Yuan Zhang, Lifang Jiang, Jiajia Li, Jian Chai, Lijun Pei, Xuejun Shang

**Affiliations:** 1 Institute of Population Research and China Center on Population Health and Development, Peking University, Beijing 100871, China; linshiqi@pku.edu.cn (S.L.); zyzyzhangyuan@pku.edu.cn (Y.Z.); lijiajia95@pku.edu.cn (J.L.); 2 National Health Commission Key Laboratory of Birth Defects Prevention, Henan Key Laboratory of Population Defects Prevention, Zhengzhou 450002, China; jiangzz66@163.com (L.J.); stopdo@163.com (J.C.); 3 Department of Andrology, Jinling Hospital, School of Medicine, Nanjing University, Nanjing 210002, China

**Keywords:** spontaneous abortion, vitamin D deficiency, socio-economic status, population-based case–control study, interactive effects

## Abstract

Background: Maternal vitamin D deficiency might generate adverse reproductive outcomes, and socio-economic inequalities in micronutrient-related diseases have often been found. This study aimed to explore the interactive effects of maternal vitamin D status and socio-economic status (SES) on risk of spontaneous abortion. Methods: A population-based case–control study was conducted including 293 women with spontaneous abortion and 498 control women in December 2009 and January, 2010 in Henan Province, China. Information on pregnancy outcomes, maternal demographic, lifestyle and exposure factors and blood samples were collected at the same time. Vitamin D deficiency was defined as 25(OH)D < 20 ng/mL. SES index was constructed with principal component analysis by aggregating women’s and their husbands’ education level and occupation, and household income and expenditure. Interactive effects were assessed on a multiplicative scale with ratio of the odds ratio (ROR). Results: Compared to those with high SES and vitamin D sufficiency, women with vitamin D deficiency and low SES index had an increased risk of spontaneous abortion (aOR: 1.99; 95% CI: 1.23–3.23). The ROR was 2.06 (95% CI: 1.04–4.10), indicating a significant positive multiplicative interaction. Conclusions: Maternal low SES may strengthen the effect of vitamin D deficiency exposure on spontaneous abortion risk in this Chinese population.

## 1. Introduction

Spontaneous abortion could generate physical, psychosocial as well as economic burdens on families and the whole society [1]. As the largest populous country, China has made remarkable strides in women and child health [2]. Yet, the incidence of spontaneous abortion has been found to be around 10~14% in China [3,4], despite rapid economic growth and improved medical services. Understanding the etiology of spontaneous abortion and finding ways to prevent it still remain crucial tasks in China.

Maternal vitamin D deficiency might generate adverse health effects on the mother as well as on the fetus [5]. Sunlight exposure and diet, including food and dietary supplements, are major sources of vitamin D in humans [6]. It is recognized that humans are physiologically capable of obtaining vitamin D through exposure to sunlight, yet the synthesis ability could be impeded by several environmental (such as latitude and prevailing weather conditions) and personal characteristics (such as age) [7], as well as some lifestyle factors (such as the use of sunscreen, working environment, physical activity and sun exposure behavior that could further reduce synthesis ability [8,9]). Therefore, a large proportion of world population, especially those living at latitudes greater than approximately 40°, should depend on dietary sources and body stores to maintain a sufficient vitamin D level all year round [10]. However, studies have indicated that pregnant women take insufficient vitamin D from their daily food [11], and do not take enough vitamin D supplements [12]. In China, there was found to be a high prevalence of vitamin D deficiency among both pregnant and unpregnant women [13,14]. Besides its classically recognized key role in bone metabolism and calcium homoeostasis, vitamin D could also promote antibacterial innate immune responses to infection, while suppressing adverse inflammatory adaptive immunity, thus playing a versatile part in the maintenance of the complex decidual immune system during pregnancy [15]. In addition, vitamin D might help extra villous trophoblast invasion during human placentation [16]. In other words, a deficiency of vitamin D could presumably endanger the maintenance of pregnancy and thus lead to spontaneous abortion. Some epidemiological studies lend support to this. A prospective cohort study of pregnant women in Denmark has explored the relationship between maternal serum 25-hydroxyvitamin D [25(OH)D] and the risk of subsequent spontaneous abortion and found that the concentration of 25(OH)D < 50 nmol/L was associated with over 2-fold increased adjusted hazard ratio (HR) for first-trimester spontaneous abortion (HR: 2.50; 95% CI: 1.10, 5.69) [17]. Another prospective cohort study in Sweden also showed that high maternal 25(OH)D level in gestational week ≤16 was negatively associated with pregnancy loss and 1 nmol/L increase in 25(OH)D had 1% lower odds of pregnancy loss (OR 0.99, 95% CI: 0.98~1.00) [18]. Considering vitamin D deficiency is very prevalent in Chinese women of childbearing age [13,19,20,21], and considering the costs created by spontaneous abortion, it is an urgent need to find whether this deficiency is one of the causes of spontaneous abortion.

A body of studies have revealed that lower SES is one of the most common risk factors associated with adverse pregnancy or neonatal outcomes, including stillbirth, neonatal mortality, congenital heart disease and spontaneous abortion [22]. Besides, consistent evidence across many countries has indicated that people in lower SES suffer more from inadequate intake and deficiency of vitamin D [23,24,25]. For example, a nationwide survey in Korea found people with ≤middle school education attainment had over 40% increased odds ratio of vitamin D deficiency compared to those with ≥high school education attainment [26]. A cross-sectional study carried out among 1250 postmenopausal Caucasian Spanish women also revealed that low socio-economic status was associated with 25-OHD insufficiency [24]. Our previous studies among women of childbearing age in Henan Province, China also found lower household income, inadequate income for expenditure and lower SES were all risk factors of vitamin D insufficiency, deficiency and severe deficiency. Possible explanations could be that people in higher SES groups have more access to nutrient supplements and more ability to understand and implement dietary guidance messages and risk-reducing dietary behaviors [23]. Therefore, there might exist a possibility that maternal vitamin D deficiency and low SES would reinforce each other to increase the risk of spontaneous abortion. The aim of the present study was thus to explore the interactive effects of maternal vitamin D status and SES on the risk of spontaneous abortion.

## 2. Materials and Methods

### 2.1. Study Design and Population

This was a population-based case–control study in Henan Province, China in December 2009 to January 2010. Lying in the central part of China (Latitude/Longitude: 31°23′ N–36°22′ N/110°21′ E–116°39′ E), Henan was home to 99.37 million people in 2020. The data were derived from the baseline dataset in the Study on Population-based Birth Defects Monitoring and Comprehensive Intervention Project in Henan Province. A multi-stage cluster sampling method was used to obtain a representative sample of women of childbearing age. Briefly, four counties were randomly selected from the 158 counties in Henan Province, China. For each county, 10 towns were randomly selected; for each town, 10 villages were randomly selected; and for each village, 10 women of childbearing age were randomly selected. Among them, 293 had spontaneous abortion within one year and were thus taken as cases in the present study; While all women who had normal liveborn babies (no birth defects, ≥37 gestational weeks and birth weight ≥ 2500 g) within the same period were taken as controls (*n* = 498).

The main aim of the present study was to evaluate the relationship of maternal SES, vitamin D status and their interaction with the risk of spontaneous abortion. The minimum sample size for the case or control group is:

$$n=\frac{2\bar{p}\bar{q}{\left(z_{\alpha }+z_{\beta }\right)}^{2}}{{\left(p_{1}-p_{0}\right)}^{2}}$$

where:

$$p_{1}=\frac{p_{0}OR}{1+p_{0}\left(OR-1\right)}$$

$$\bar{p}=0.5\times \left(p_{1}+p_{0}\right)$$

$$\bar{q}=1-\bar{p}$$

For 95% confidence intervals and α=5%, thus $z_{\alpha }=1.96$; for 90% power of test and β=10%, thus $z_{\beta }=1.28$; $p_{1}$: the proportion of exposure in cases; $p_{0}$: the proportion of vitamin D deficiency exposure in controls, which was set at 50% according to previous literature [14,20]; OR: odds ratio of the relationship between exposure and the outcomes which was set at 2.0.

Therefore, the minimum sample size of one group was 184. Our sample size (*N* = 293 + 498 = 791) was thus good enough to discover the relationships of interest to us.

### 2.2. Collection of Data and Blood Sample

Trained healthcare workers conducted face-to-face interviews with participants and their families to collect information on the women’s and their husbands’ demographic and social economic characteristics, history of diseases and treatment, eating habits and the frequency of dietary and nutrient intake, behavioral factors, and the utilization of public health services.

For each participant, a fasting venous blood sample (8 mL) was also collected at interview by professional healthcare workers. The collection time of blood samples was all in winter (December 2009 and January 2010). The sample was prepared by centrifugation and stored at −80 °C at Peking University until analysis.

The study protocol was reviewed and approved by the Institutional Review Board of Peking University Health Science Center, and written informed consent was obtained from all subjects before completing the questionnaire and collection of blood samples at the time of the baseline survey.

### 2.3. Definition of Cases and Controls

Cases were women with spontaneous abortion, which were defined as clinically recognized pregnancy loss before 28 gestational weeks. Controls were women with healthy liveborn babies without birth defects, preterm birth (≥37 gestational weeks) or low birth weight (birth weight ≥ 2500 g), the control outcomes were obtained by following the women’s pregnancy outcomes. All the pregnancy information was self-reported by participants during face-to-face interviews implemented by well-trained health care workers.

### 2.4. Definition of Vitamin D Deficiency

Serum 25-hydroxyvitamin D (25(OH)D) concentration in serum samples were analyzed by the lab at Beijing Mass Spectrometry Medical Research Ltd., Beijing, China, a third-party inspection organization that strictly follows state of the art quality assurance procedures of ISO15189. A High-performance Liquid Chromatography-Tandem Mass Spectrometry (HPLC-MS/MS, Ultimate3000–API 3200 Q TRAP) method was adopted to quantitatively determine the 25(OH)D concentrations. The data on 25(OH)D included in this manuscript have already been published elsewhere [14]. Vitamin D deficiency was defined as <20 ng/mL and sufficiency as ≥20 ng/mL [27].

### 2.5. Definitions of Socio-Economic Status

In our study, an SES index was constructed by aggregating six indicators of SES through principal component analysis: women’s education level, their husbands’ education level, women’s occupation, their husbands’ occupation, household annual income and whether their annual income was enough for expenditure. Women’s or their husbands’ educational level were grouped as “high school or above” and “junior high school or below”; Women’s and their husbands’ occupation were divided into “unemployed or farmers” and “other occupations”; Household annual income was also divided into two groups with 10,000 RMB (RMB is the official currency of China) as the cut-off point; and household income for expenditure came from the question “whether your family have enough income for expenditure in your daily life?” and was grouped into “surplus” and “inadequate or deficit”. The principal component analysis was taken to construct the SES index [28]. The co-variance matrix was used, and the Bartlett’s test of sphericity was statistically significant (*p* < 0.001), indicating that it was suitable for the use of the principal component analysis. Generally, the number of principal components extracted to construct the index can be defined by the researcher [28], yet here the first principal component was taken as a measure of SES index as previous studies have found that the first principal component was a good measure of socio-economic status [29]. The SES index was then divided into high and low SES subgroups with the 33.3 percentile as the cut-off points, i.e., the highest 66.6% SES index was grouped as “high SES” and the rest as “low SES”.

### 2.6. Covariates

The potential covariates were selected on the basis of previous knowledge. There were two types of potential covariates that were considered in our study: potential confounders of the relationship between risk of spontaneous abortion and SES or vitamin D status, including age, BMI, history of chronic diseases, passive smoking, alcohol intake and dietary intake [30,31,32,33]. Notably, personal smoking is often seen as an important confounder. Yet, in our dataset, only one participant (0.1%) had personal smoking during pregnancy. Considering common situations in real lives that women seldom smoke in China and reports of other studies that the prevalence of tobacco smoking is traditionally low among Chinese women, about 6.4% in 2003 [34], the figure in our study is reasonable. Therefore, we did not include personal smoking here. Yet, since non-smokers might also be exposed to tobacco smokes and passive smoking has also been found to be associated with vitamin D deficiency [35], we included passive smoking here. Another class of covariates were factors that might not be confounders but can also influence the risk of spontaneous abortion, which, when included in the final multivariate model, can help increase the model’s explanatory power, such as, utilization of health services and physical exercise [33,36,37,38]. As the month of blood sampling is closely associated with sunlight exposure and thus could be a very important reason affecting vitamin D level, we also regarded it as a potential covariate.

BMI was calculated by weight/height^2^ and was grouped into <24 kg/m^2^ as normal weight or underweight, ≥24 kg/m^2^ to <28 kg/m^2^ as overweight and ≥28 kg/m^2^ as obesity based on body characteristics of Chinese population [39]. Women’s weight and height data were recorded by professional health-care workers in physical examinations; History of chronic diseases referred to having been diagnosed with any one of the following diseases: anemia, hypertension, hyperlipemia, heart disease, diabetes, hyperglycemia, thyroid diseases, phenylketonuria, epilepsy, asthma, chronic renal diseases, systematic lupus erythematosus, rheumatic arthritis, deep vein thrombosis, cancer, depression or anxiety and schizophrenia.

Nutritional factors included nutritional supplement, vitamin D supplement, meat intake, fish intake, eggs intake, milk or dietary products, beans and soy product intake and vegetable intake during the past year. Vitamin D supplement included supplementation of vitamin D, cod-liver oil or multivitamins containing vitamin D. Nutritional supplement included any supplement of the following nutrients: vitamin A, multivitamin B, vitamin B1, vitamin B2, vitamin B6, vitamin B12, vitamin C, vitamin E, cod-liver oil or vitamin D, iron preparations, calcium tablets or zinc. Food intake referred to average frequencies of the food intake during the past year.

Behavioral factors included alcohol consumption, passive smoking and physical exercises during the past year. Having passive smoking referred to “passively inhaling cigarette smoke by smokers around you for more than 15 min every day”; taking physical exercises was defined as taking indoor or outdoor exercises (including walking, running, ball games, Tai Chi or other health-promoting physical exercises, swimming and other sports) once a week for more than 30 min per time.

Utilization of health services included accepting community maternal-infant health promotion services and accepting physical examination during the past year. Accepting community maternal-infant health promotion services meant that women received knowledge of maternal and child health, such as how to prepare for pregnancy, from health service institutions during the past year.

### 2.7. Statistical Analysis

Univariate analysis was conducted to test the differences of demographic characteristics, SES, nutritional and behavioral variables and utilization of public health services through $\chi^{2}$ test. The statistical significant ones (*p* < 0.05) were then regarded as covariates to be included in later multivariate models.

In a multivariate analysis, multilevel logistic regression analyses were performed to reflect the multi-stage sampling selection. A random intercept for villages was thus included in regression models to account for clustering within villages. We first built a model to explore the relationship between vitamin D status, SES and spontaneous abortion by adjusting for covariates.

Another multi-level logistic model was then used to evaluate the interactive effects. Subjects were divided into four groups: (1) women with high SES index and sufficient vitamin D; (2) those with high SES index but deficient vitamin D; (3) those with sufficient vitamin D but low SES index; (4) those with low SES index and deficient vitamin D. The four groups were then entered simultaneously into the multivariate, multilevel logistic regression model with the first group as the reference group and with adjustments of significant variables in univariate analyses. The interactive effects can be reflected by the adjusted OR of group (4).

The equation of the regression model was:

$$Logit\left(odds\right)=\gamma_{00}+u_{0j}+\beta_{1j}X_{VDD}+\beta_{2j}X_{LSES}+\beta_{3j}X_{LSES+VDD}+B_{j}C_{kj}+\varepsilon_{ij}$$

where $odds=P\left(Y_{i}=1\right)/\left(1-P\left(Y_{i}=1\right)\right)$, $Y_{i}=1$ meant the pregnancy outcome was a spontaneous abortion case; $\gamma_{00}$ was the fixed intercept, while $u_{0j}$ was the deviation of the cluster-specific intercept from the fixed intercept; $X_{VDD}\;$represented the vitamin D deficiency and high SES group; $X_{LSES}$ represented low SES and vitamin D sufficiency group; $X_{LSES+VDD}$ represented the low SES and vitamin D deficiency group; $C_{kj}$ were variables adjusted in the model; and $\beta_{1j}$, $\beta_{2j}$, $\beta_{3j}$ and $B_{j}$ were the regression coefficients; $\varepsilon_{ij}$ was the error term.

The extent to which the joint effects of women’s vitamin D deficiency and low SES index exceeded the effects of each factor considered individually was assessed on a multiplicative model with a ratio of the odds ratio (ROR) [40]:

$$ROR=OR_{11}/\left(OR_{10}\times OR_{01}\right)$$

where $OR_{11}$, $OR_{10}$ and $OR_{01}$ were odds ratio of group (4), (3) and (2). ROR > 1 and *p* < 0.05 indicated a significant positive interaction between women’s vitamin D deficiency and low SES.

## 3. Results

### 3.1. Basic Characteristics of Participants and Univariate Analysis

Table 1 presents the differences between demographic, socio-economic, nutritional, behavioral and vitamin D status between spontaneous abortion cases and controls. The majority of mothers in our study were over 28 years old (61.82%), with middle school education or lower (76.30%), unemployed or working on household farms (80.66%) and most fathers were also of middle school or lower education level (74.21%) and had jobs other than farmers (61.32%). Over 60% of families had an annual income ≥10,000 RMB, but 66.20% thought their income was not balanced. The majority underwent blood sampling in Dec 2007 (*n* = 665, 84.07%). It is noteworthy that although nearly half of participants were in a state of vitamin D deficiency (*n* = 389, 49.18%), only a very limited number of them had taken vitamin D supplements (*n* = 74, 9.36%) during the past year, among whom only 19 were in the low SES group.

Univariate analysis showed spontaneous abortion cases tended to suffer more from vitamin D deficiency and lower SES. The rate of vitamin D deficiency was higher in those with lower SES compared to their counterparts with higher SES (53.36% vs. 46.80%), though the difference was not significant (*p* = 0.078). There existed significant differences between spontaneous abortion and controls in terms of BMI, history of chronic diseases, passive smoking, meat intake, milk intake, and the acceptance of community maternal-infant health promotion services. These factors were then taken as covariates in the later multivariate analysis. (Table 1)

### 3.2. Independent Association between Maternal Vitamin D Deficiency and Risk of Spontaneous Abortion

We first examined the independent association between vitamin D status and the risk of spontaneous abortion by multi-level, multivariate logistic regression model analysis. It was found that maternal vitamin D deficiency was associated with an increased risk of spontaneous abortion (OR = 1.47, 95% CI: 1.03–2.09) after adjusting for BMI, SES index, history of chronic diseases, vitamin D status, passive smoking, meat intake, milk intake, and accepting community maternal-new-born health promotion services.

### 3.3. Independent Association between SES and Risk of Spontaneous Abortion

The independent association between SES and the risk of spontaneous abortion was then examined, also using multi-level, multivariate logistic regression model analysis. The results showed that lower SES had 28% higher odds of spontaneous abortion, though the association was not significant (95% CI: 0.90–1.82, *p* = 0.163) after adjusting for BMI, vitamin D status, history of chronic diseases, vitamin D status, passive smoking, meat intake, milk intake, and accepting community maternal-infant health promotion services.

### 3.4. Interactive Effects of Maternal Vitamin D Status and SES on Spontaneous Abortion

Table 2 presents the results of the combined and interactive effects between maternal vitamin D status and socio-economic status on the risk of spontaneous abortion. Compared to the high SES and maternal vitamin D sufficiency group, the group with both low SES index and maternal vitamin D deficiency had an increased risk of spontaneous abortion (aOR: 1.99; 95% CI: 1.23–3.23) after adjusting for BMI, history of chronic diseases, passive smoking, meat intake, milk intake, and acceptance of community maternal-new-born health promotion services. The ROR was 2.06 (95% CI: 1.04–4.10), indicating a significant positive multiplicative interaction.

## 4. Discussion

There are two main findings in our study. Firstly, our findings indicated that women with vitamin D deficiency were more likely to have experienced spontaneous abortion. This result agrees with previous studies suggesting vitamin D’s vital role in successful pregnancy and fetal growth: vitamin D plays a role in immunoregulation and trophoblast invasion [15,16], both of which are key to implantation [32]; vitamin D is also important for a healthy pregnancy, as it is related to calcium metabolism in the myometrium, has a direct role in the production of antimicrobial peptides and may help prevent infection during pregnancy [41]. Some past epidemiological studies have also suggested the potential impacts of maternal vitamin D deficiency on adverse pregnancy outcomes [17,18], yet few have focused on Chinese women. Our study thus revealed this association in a Chinese population. Although the Chinese Pediatric Society has issued guidance advising women to monitor their serum 25(OH)D level during the periconceptional period and to take 3000~5000 IU/day of vitamin D supplements if they have vitamin D deficiency [42], the vitamin D status and supplementation among women of childbearing age, as well as maternal vitamin D policies or supplementation/dietary practices, have not made much progress. Most measures or community health promotions regarding vitamin D supplementation are focused on the elderly or children, while adults including pregnant women were given less attention; antenatal care does stress the importance of nutrition and balanced diet, and nutrient supplementation is beginning to be recognised more and more by pregnant women [43], yet there still exists a gap between health knowledge and behavior. Some surveys have shown that only 1.4% of pregnant women take enough vitamin D from their diet and fewer than 50% of them take vitamin D supplements (including multivitamin supplements) [12,43,44]. In fact, many national or local surveys have shown that the majority of Chinese women at childbearing age, both pregnant and unpregnant, are in a constant state of vitamin D deficiency [13,19,20,21]. As reported by one national study, vitamin D deficiency has persisted and has even become more severe during the past decade (the deficiency rate increased from 74.83% to 87.43% from 2010–2012 to 2015–2017) [19]. This research is consonant with our findings that only 74 (9.36%) among all participants took vitamin D supplements, while nearly half of them were under vitamin D deficiency. Therefore, although our study was based on data from over ten years ago, our findings still have implications for future antenatal care policies. On the other hand, in China, vitamin D is often supplemented together with calcium or other vitamins, as it was also shown in our study that 72 out of the 74 participants took both vitamin D supplements and calcium supplements (including calcium supplements alone and multivitamin-mineral supplements). However, the status, “safe range” and requirements of nutrition might vary across different people, and thus a dose that is beneficial or sufficient for some subgroups in the population may be harmful to or insufficient for others [45]. In particular, excessive vitamin D leads to hypercalcaemia and hypercalciuria, which are further associated with renal and kidney stones [46]. A meta-analysis has suggested that supplementation with vitamin D and calcium during pregnancy might increase the risk of preterm birth (RR = 1.52, 95% CI: 1.01~2.28) [47]. Therefore, the overconsumption of calcium and vitamin D should be cautioned and natural sources of calcium such as milk and its products can be considered for some subgroups. In summary, not only vitamin D supplementation but also personalized nutrition guidance should be stressed in antenatal care to reduce adverse pregnancy outcomes.

Secondly, as our study discovered, women with vitamin D deficiency and low SES were more likely to have experienced spontaneous abortion compared to those of vitamin D sufficiency and high SES. This finding is also consonant with previous studies that have found suboptimal maternal vitamin D status and low education level as determinants of small-for-gestational-age birth weight [48]. Women in low SES have often been observed to have malnutrition, insufficient health knowledge, unhealthy lifestyles, poor living environment and limited access to health services [49,50,51]. All of the aforementioned factors might result in a worse baseline health status for themselves and their babies, as reflected by some research findings that women in low SES are more susceptible to stillbirth and perinatal mortality [52,53]. Besides, as indicated by many previous studies, women of lower education, lower income, unemployment status are more likely to suffer from vitamin D deficiency [54,55,56]. Women in low SES often have higher exposure to active or passive smoking [57], and nicotine, through several pathways such as inhibiting vitamin D-parathyroid hormone axis, decreases serum 25(OH)D and 1,25(OH)2D levels, vitamin D intake from diet, and the cutaneous production of vitamin D through skin aging [58]. Considering it has been revealed that the relationship between maternal serum vitamin D level and adverse pregnancy outcomes were non-linear and that lower vitamin D level had higher risk of adverse pregnancy outcomes [59], the joint effects of low SES and low vitamin D status could worsen pregnancy outcomes. In addition, suffering from vitamin D deficiency, women from low SES might be less prone to take vitamin D supplementation [60]. As our study found out, among the 74 participants taking vitamin D supplements, only 19 were from low SES group. Identifying a strengthened negative influence of vitamin D deficiency by low SES, our findings revealed nutritional statuses and health disparities across the SES population.

The main strength of the present study was that a population-based case–control study was conducted with a large representative sample size. Participants were chosen through the multi-stage cluster sampling method, thus minimizing selection bias. As Henan Province is among the most populous province, lying in the central part of China, participants from Henan Province could be good representations of the average status of the Chinese population. Moreover, by constructing an SES index, we reduced potential bias that might be brought about by a single indicator, thus improving the test power. Instead of assigning scores to different dimensions of SES to construct the index, the principal component analysis was taken here, thus increasing credibility and reliability.

Our study was not without its limitations. Firstly, we assumed a relative stability of the serum 25(OH)D and thus regarded the measurement after the pregnancy outcomes as an estimate of vitamin D exposure during pregnancy. Although this is a very common assumption in previous studies [61], vitamin D levels can be influenced by a number of factors, including seasonal changes and weight status, thus there are some recent studies questioning this practice in long-lasting observational studies [62]. In particular, the blood samples in our study were all collected during winter when sunlight and temperature was lowest throughout the year, therefore serum 25(OH)D levels might be underestimated. Yet, in our study, the interval between pregnancy and serum 25(OH)D measurement time was not overly long (about one year), and the effects of vitamin D deficiency on spontaneous abortion are more likely to be cumulative ones rather than sporadic ones. Currently, neither the government nor non-profit organizations have implemented any strategies to improve vitamin D supplementation in women of childbearing age, nor is there any evidence showing that women who have suffered pregnancy loss tend to take higher vitamin D supplements. In fact, as some previous studies have shown, only a very small proportion of women took vitamin D supplement (8.97%, very close to the 9.36% in our study) in 2010–2012 [19], and vitamin D supplementation was even lower in central China (our study setting, Henan Province, is among the largest provinces in central China) than that in southern China. Therefore, it is reasonable to assume that our measurement of vitamin D levels approximated cumulative vitamin D exposure during pregnancy. Secondly, diet could be an important confounder in the relationship between vitamin D status and spontaneous abortion, yet the method we adopted to assess dietary intake frequency could not measure the exact vitamin D intake. However, naturally occurring vitamin D is rare in foods, and pregnant women’s dietary intake of vitamin D has constantly been found to contribute very little to serum vitamin D levels [9] and thus cannot satisfy the recommended vitamin D intake in many populations including China [11,63]. Future studies can consider establishing a prospective cohort study and tracking changes of serum 25(OH)D levels for several times during pregnancy to help establish causal association between SES and vitamin D deficiency on spontaneous abortion.

## 5. Conclusions

In summary, vitamin D deficiency might be associated with a higher risk of spontaneous abortion. Additionally, low SES can reinforce the effects of vitamin D deficiency on the risk of spontaneous abortion. Our study thus calls for promoting vitamin D fortified food or supplements, health education and health monitoring among women of childbearing age. Moreover, our findings might stimulate further research into the health disparities across SES groups in maternal nutritional status and pregnancy outcomes, and also suggests that more attention be paid to the nutritional status and health behaviors of women in low SES to reduce the occurrence of spontaneous abortion.

## Figures and Tables

**Table 1:** Demographic, socio-economic, nutritional, behavioral and vitamin D status differences between spontaneous abortion cases and controls.

Variable	Cases (*n* = 293)	Controls (*n* = 498)	$\chi^{2}$	*p*
	N(%)	N(%)		
Age				
<28	110(37.54)	192(38.55)		
28–	183(62.46)	306(61.45)	0.08	0.777
BMI (kg/m^2^)				
<24	170(58.02)	325(65.26)		
24–28	79(26.96)	126(25.30)		
28–	44(15.02)	47(9.44)	6.73	0.035
History of chronic diseases				
No	243(82.94)	450(90.36)		
Yes	50(17.06)	48(9.64)	9.37	0.002
Socio-economic status				
Women’s education				
High school or above	61(20.82)	126(25.40)		
Junior high or below	232(79.18)	370(74.60)	2.14	0.143
Husband’s education				
High school or above	74(25.26)	130(26.10)		
Junior high or below	219(74.74)	368(73.90)	0.07	0.792
Women’s occupation				
Others	55(18.77)	98(19.68)		
Unemployed or famers	238(81.23)	400(80.32)	0.10	0.755
Husband’s occupation				
Others	193(66.55)	289(58.27)		
Unemployed or famers	97(33.45)	207(41.73)	5.30	0.021
Household annual income (RMB *)				
≥10,000	172(58.70)	307(61.90)		
<10,000	121(41.30)	189(38.10)	0.79	0.375
Household income for expenditure				
Surplus	77(26.28)	190(38.23)		
Inadequate or deficit	216(73.72)	307(61.77)	11.76	0.001
SES index				
High	169(58.28)	331(67.14)		
Low	121(41.72)	162(32.86)	6.22	0.013
Nutritional factors				
Nutritional supplement				
No	216(73.72)	382(76.71)		
Yes	77(26.28)	116(23.29)	0.89	0.345
Vitamin D supplement ^†^				
No	261(89.08)	456(91.57)		
Yes	32(10.92)	42(8.43)	1.35	0.246
Meat intake				
≥once per week	144(49.15)	286(57.55)		
<once per week	149(50.85)	211(42.45)	5.24	0.002
Fish intake				
≥once per month	51(17.41)	111(22.33)		
<once per month	242(82.59)	386(77.67)	2.75	0.10
Eggs intake				
Everyday	92(31.40)	157(31.59)		
4–6 times per week	60(20.48)	131(26.36)		
≤3 times per week	141(48.12)	209(42.05)	4.17	0.124
Milk or dairy products intake				
≥4 times per week	49(16.72)	100(20.12)		
<4 times per week but at least once per month	70(23.89)	160(32.19)		
Almost never	174(59.39)	237(47.69)	10.34	0.006
Beans and soy products intake				
Everyday	85(29.01)	131(26.36)		
4–6 times per week	53(18.09)	106(21.33)		
1–3 times per week	72(24.57)	143(28.77)		
<once per week	83(28.33)	117(23.54)	4.30	0.231
Vegetables and fruits intake				
Everyday	213(72.70)	354(71.23)		
<once per day	80(27.30)	143(28.77)	0.196	0.658
Behavioral factors				
Passive smoking				
No	97(33.11)	215(43.17)		
Yes	196(66.89)	283(56.83)	7.83	0.005
Physical exercise				
No	251(85.67)	424(85.48)		
Yes	42(14.33)	72(14.52)	0.01	0.944
Utilization of health services				
Accept community maternal-newborn health promotion services				
No	163(55.82)	228(46.25)		
Yes	129(44.18)	265(53.75)	6.72	0.010
Accept physical examination during the past year				
No	186(63.48)	293(59.43)		
Yes	107(36.52)	200(40.57)	1.27	0.261
Vitamin D status				
Sufficient	129(44.03)	273(54.82)		
Deficient	163(55.97)	225(45.18)	8.60	0.003
Sampling month				
January	42(14.33)	84(16.87)		
February	251(85.67)	414(83.13)	0.88	0.347

SES index, socio-economic status index; * RMB is the Chinses official currency; **^†^** vitamin D supplements including supplementation of vitamin D, cod-liver oil or multivitamins containing vitamin D.

**Table 2:** The interactive effects between vitamin D status and socio-economic status on the risk of spontaneous abortion.

SES	Vitamin D status	Controls	Cases	aOR(95% CI) *	ROR(95% CI)
High	Sufficient	181	85	1.00	
Low	Sufficient	89	43	0.86(0.52~1.44)	
High	Deficient	150	84	1.12(0.72~1.73)	
Low	Deficient	73	78	1.99(1.23~3.23)	2.06(1.04~4.10) ^†^

SES index, socio-economic status index; ROR: ratio of odds ratio. * Multi-level logistic regression models adjusted for body mass index, history of chronic diseases, passive smoking, meat intake, milk intake and accepting community maternal-new-born health promotion services. ^†^ $P_{interaction}<0.05$

## Data Availability

The data are available from the corresponding author by reasonable request.
